# Malignant Mixed Mullerian Tumor (Carcinosarcoma) in the Female Genital Tract: A Retrospective Study From a Single Center in North India

**DOI:** 10.7759/cureus.72317

**Published:** 2024-10-24

**Authors:** Shivanjali Raghuvanshi, Nidhi Gupta, Kusum Yadav, Sneha Chaudhary, Mithilesh, Manish Kumar, Akanksha Sharma

**Affiliations:** 1 Pathology, King George's Medical University, Lucknow, IND; 2 Pathology, Dr. KNS Memorial Institute of Medical Sciences, Lucknow, IND; 3 Pathology, Dr. Ram Manohar Lohia Institute of Medical Sciences, Lucknow, IND

**Keywords:** carcinosarcoma, malignant mixed mullerian tumor, mmt, ovarian malignancy, uterine malignancy

## Abstract

Background

Carcinosarcoma (malignant mixed Mullerian tumor (MMT)) is a rare tumor of the uterus, even rarer in the ovary. Presenting symptoms are indistinguishable from high-grade uterine endometrium carcinoma. Histologically, tumors are biphasic, composed of both epithelial and mesenchymal (sarcomatous) components. The diagnosis of carcinosarcoma presents a challenge due to the need to exclude commonly occurring mimickers, necessitating the application of a broad panel of immunohistochemistry markers.

Material and methods

This retrospective descriptive study of malignant MMT was conducted over a three-year period (August 2021 to July 2024). A total of six (three ovarian and three uterine) cases diagnosed as malignant MMT were included based on morphology and confirmation by expressing positivity of cytokeratin (CK) and vimentin. All the demographic, clinical, and radiological data, treatment protocols, and survival data were obtained from the medical record department. Dako^® ^(Agilent Diagnostics and Genomics Group, Santa Clara, California) antibodies were used for immunohistochemical (IHC) analysis with 1:100 dilutions.

Results

The mean age of presentation was 51.5 years (35-65 years). The primary tumor site was the uterus in four cases, bilateral ovaries in one case, and unilateral ovaries in one case. Metastasis was seen in two cases. Microscopically, the tumor was biphasic, displaying high-grade carcinomatous and sarcomatous components. Pan-CK was positive in the epithelial component of the tumor, while vimentin was positive in high-grade sarcomatous components. Epithelial membrane antigen (EMA) was positive in three cases, while myogenic was positive in heterologous rhabdomyosarcomatous component in one case. The mean ki-67 was approximately 52% (30%-80%).

Conclusions

Carcinosarcomas are highly aggressive tumors commonly occurring in elderly females. Uterine carcinosarcomas are rare, and ovarian carcinosarcomas are even rarer. Clinical and histological features resemble high-grade endometrial carcinoma, necessitating an extensive IHC panel to narrow down differentials and confirm the diagnosis. This study highlights the histological and IHC features and emphasizes that early diagnosis plays a crucial role in disease management.

## Introduction

Carcinosarcoma, also known as malignant mixed Mullerian tumor (malignant MMT), is an uncommon uterine tumor that accounts for less than 5% of uterine malignancies and typically occurs in elderly women [1,2]. Abnormal uterine bleeding, abdominal pain, and a rapidly growing mass are the primary symptoms, which are indistinguishable from those of high-grade endometrial carcinoma [3]. Similar to endometrial carcinoma, commonly occurring risk factors are nulliparity, estrogen or tamoxifen therapy, and obesity [2,4]. Studies demonstrated that advancing age, histology, non-White race, and advanced tumor stage are independent risk factors for poor prognosis [5]. Due to its aggressive nature, metastasis is commonly seen in the lymph nodes, lungs, and various other locations [6].

Ovarian carcinosarcomas are even rarer and account for only <1%-2% of all malignant ovarian tumors [7]. Histologically, carcinosarcomas are biphasic tumors displaying morphology of both epithelial and mesenchymal components resulting from epithelial-mesenchymal transition (EMT) [8]. Molecular studies showed a common monoclonal origin for most of the carcinosarcomas and their derivation from epithelial to evolution of sarcomatous components [9-11]. Although various genetic mutations are seen associated with the pathogenesis of carcinosarcoma, the most common are *TP-53* (91%), followed by *PIK3CA* (35%) [9]. Since clinical features alone cannot diagnose malignancy, imaging studies, histopathological examination, and confirmation by immunohistochemical (IHC) evaluation are necessary.

Carcinosarcoma is a unique gynecological malignancy that poses diagnostic and therapeutic challenges in the field of oncology; hence, a detailed reporting of clinical, radiological, and histopathological features, treatment protocol, and overall survival outcomes is well-intentioned. We hereby present a retrospective study of six reported cases of carcinosarcoma of the female genital tract (ovarian and uterine) from a three-year period of study. The primary objective of the study is to evaluate the clinical presentation, histopathological features, and treatment outcomes of malignant MMTs of the female genital tract in a North Indian population. The secondary objective is to assess the prognosis and survival status associated with these tumors and identify factors influencing outcomes based on the current treatment strategy.

## Materials and methods

This retrospective descriptive study of MMT was done within a three-year duration (August 2021 to July 2024) with cases diagnosed at the Department of Pathology, King George's Medical University, Lucknow, a tertiary care center in North India. A total of six cases diagnosed as MMT were included based on morphology and confirmation by expressing positivity of CK and vimentin IHC markers. Cases negative for cytokeratin (CK) and vimentin and positive for any other differentiation markers such as leukocyte common antigen (LCA), WT-1, synaptophysin, chromogranin, HMB45, estrogen receptor (ER), and progesterone receptor (PR) were excluded from the study.

All demographic and clinical data were collected directly from the archive or medical record department after obtaining patient consent. Data were manually and electronically collected using unique identification number (UHID) and bed head ticket (BHT) files. Treatment and survival data were obtained with the collaboration of the Department of Medical Oncology (Table 1).

**Table 1:** Demographic and clinical characteristics of the cases enrolled and their disease progression. *Symptoms progressed rapidly over the course of days, weeks, or months. **Symptoms progressed gradually over a span of months or years.

Case No.	Age (Years)/Sex (M/F)	Specimen Received	Tumor Site	Tumor Size (cm)	Presenting Symptoms	Duration of Symptoms (Months)	Progression	Distant Metastasis
1	35/F	Uterus with cervix with B/L Salpingo-oophorectomy	Ovary with uterus	4x3.3x3 Rt ovary replaced by tumor	Abnormal uterine bleeding	2 months	Rapid*	Yes
2	62/F	Hysterectomy specimen	Uterus	5x4x3.5 Reaching up to the cervix	Post-menopausal bleeding	5 months	Rapid	No
3	52/F	Uterus with cervix with B/L salpingo-oophorectomy	B/L ovary	B/L ovaries were replaced by tumor	Pain abdomen	2 months	Rapid	No
4	40/F	Peritoneal fluid, Peritoneal biopsy, B/L salpingo-oophorectomy	Unilateral ovary with peritoneal metastasis	7x5x3.6 Adhere to omentum and peritoneum	Heaviness in the lower abdomen with ascites	3 months	Rapid	Yes
5	55/F	Hysterectomy specimen	Uterus	3.5x3x3	Post-menopausal bleeding	5 months	Slow**	No
6	65/F	Hysterectomy specimen	Uterus	2.8x2x2	Post-menopausal bleeding	4 months	Slow	No

All the collected data were compiled in Microsoft Excel (Microsoft Corporation, Redmond, Washington) sheet. The necessary statistical analysis was performed using IBM SPSS Statistics for Windows, Version 27 (Released 2020; IBM Corp., Armonk, New York). Paraffin-embedded blocks were retrieved from the departmental archive, and routine hematoxylin and eosin (H&E), Giemsa, and periodic acid-Schiff (PAS) staining were applied. Extended IHC panels were done to narrow down the differentials and confirm the microscopic findings (Table 2). For IHC staining, deparaffinized sections were cut at 3-4 μm thickness. Antigen retrieval was done by placing sections in the microwave in tris(hydroxymethyl)aminomethane-ethylenediaminetetraacetic acid (Tris-EDTA) (Tris 1.21 g, EDTA 0.37 g, Tween 20 500 µL) buffer (pH 9.0)/citrate buffer (pH 6.0) for 98°C for 25 minutes) and then cooled to room temperature. Endogenous peroxidase activity was blocked by Dako® (Agilent Diagnostics and Genomics Group, Santa Clara, California) peroxidase blocking reagent for 10 minutes, then the primary antibody was added. Sections were treated with excess buffer, wiped off, and covered with link antibody (secondary) for 30 minutes at room temperature. Slides were rinsed two times with TRIS buffer for 5 minutes each. We used the Dako® antibody with a dilution of 1:100 as a secondary antibody.

**Table 2:** Histological types, immunohistochemistry profile of the tumor, and treatment protocol with survival outcomes of cases enrolled. IHC: immunohistochemistry; FIGO: International Federation of Gynecology and Obstetrics; EMA: epithelial membrane antigen; LCA: leukocyte common antigen; CK: cytokeratin.

Case No.	Histological Type	Serum Marker (CA-125)	IHC (Positive)	IHC (Negative)	Treatment Received	Pathological Staging (FIGO 2018)	Survival Outcome
1	Carcinosarcoma of the right ovary metastasizing to the uterus	Raised	CK, Vimentin, EMA	Chromogranin, synaptophysin, HMB45, WT-1	Surgery & was planned for chemotherapy	IV	Pt. died after 15 days of diagnosis
2	Uterine carcinosarcoma with heterologous rhabdomyosarcomatous component	-	CK, Vimentin, Myogenic	WT-1, chromogranin, synaptophysin,	Surgery with chemotherapy	II	Pt. alive after ~3 years of diagnosis
3	Serous papillary cystadenocarcinoma of right ovary and carcinosarcoma of left ovary	Raised	WT-1, CK, Vimentin	LCA, synaptophysin chromogranin HMB45, CD68	Surgery with chemo with radiotherapy	III	Pt. died after 6 months of diagnosis
4	Carcinosarcoma of right ovary	Raised	Vimentin, CK, CA-125	WT-1, chromogranin, synaptophysin	Surgery with chemo with radiotherapy	IV	Pt. died after 11 months of diagnosis
5	Uterine carcinosarcoma	-	CK, Vimentin, EMA	Chromogranin, synaptophysin, HMB45, WT-1	Surgery with follow-up	IB	Pt. alive after ~2years & 8 months of diagnosis
6	Uterine carcinosarcoma	Raised	CK, Vimentin, EMA	LCA, CD68 synaptophysin, HMB45, WT-1	Surgery with follow-up	IB	Pt. alive after 1 year of diagnosis

## Results

Clinical and demographic findings

The mean age of presentation was 51.5 years (35-65 years). Out of six cases, three underwent total abdominal hysterectomy, while the remaining three had total abdominal hysterectomy with bilateral salpingo-oophorectomy. The primary tumor site in three cases was the uterus, the bilateral ovaries in one case (Case 3), and the unilateral ovary in two cases (Cases 1 and 4). Metastasis was observed in two cases (Cases 1 and 4), from the ovary to the uterus in Case 1 and from an ovarian tumor to peritoneal metastasis in Case 4. Preaortic, paraaortic, and pelvic lymph nodes were also involved in both cases. The most common clinical feature was postmenopausal bleeding, followed by abdominal pain and lower abdomen heaviness. The mean duration of clinical symptoms was 3.5 months, ranging from two to five months. Most of the cases showed aggressive behavior; however, Cases 5 and 6 were slow-growing (Table 1). Distant metastasis was unusual; however, in Case 1, the tumor metastasized into the ipsilateral ovary and peritoneum, and in Case 4, the tumor locally spread from the ovary to the peritoneum and omentum.

Radiological findings

**Figure 1:**
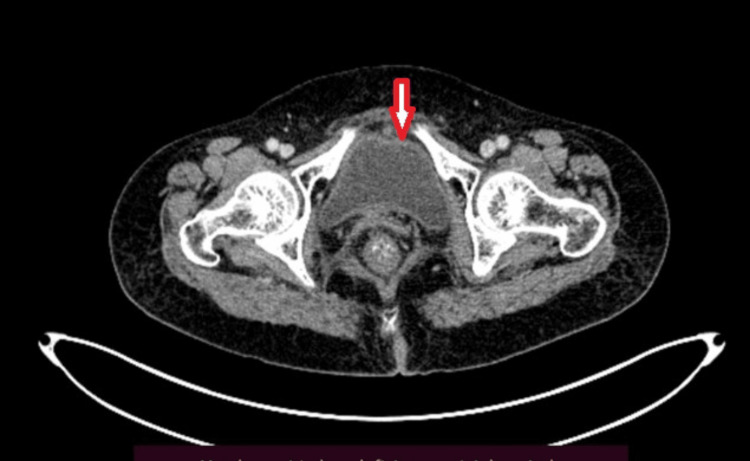
The CT image of the uterus in Case 2 shows an irregular, thickened uterus.

Radiological imaging findings are nonspecific to carcinosarcoma and cannot be distinguished from other high-grade endometrial carcinomas. Ultrasonography showed a relatively hypoechoic mass or heterogeneous thickening of the endometrium and expansion of the endometrial canal in the early stages (Stages I and II) of tumors. CT findings show an ill-defined, irregular hypoechoic mass along with dilation of the endometrial canal in most cases (Case 2) (Figure 1).

**Figure 2:**
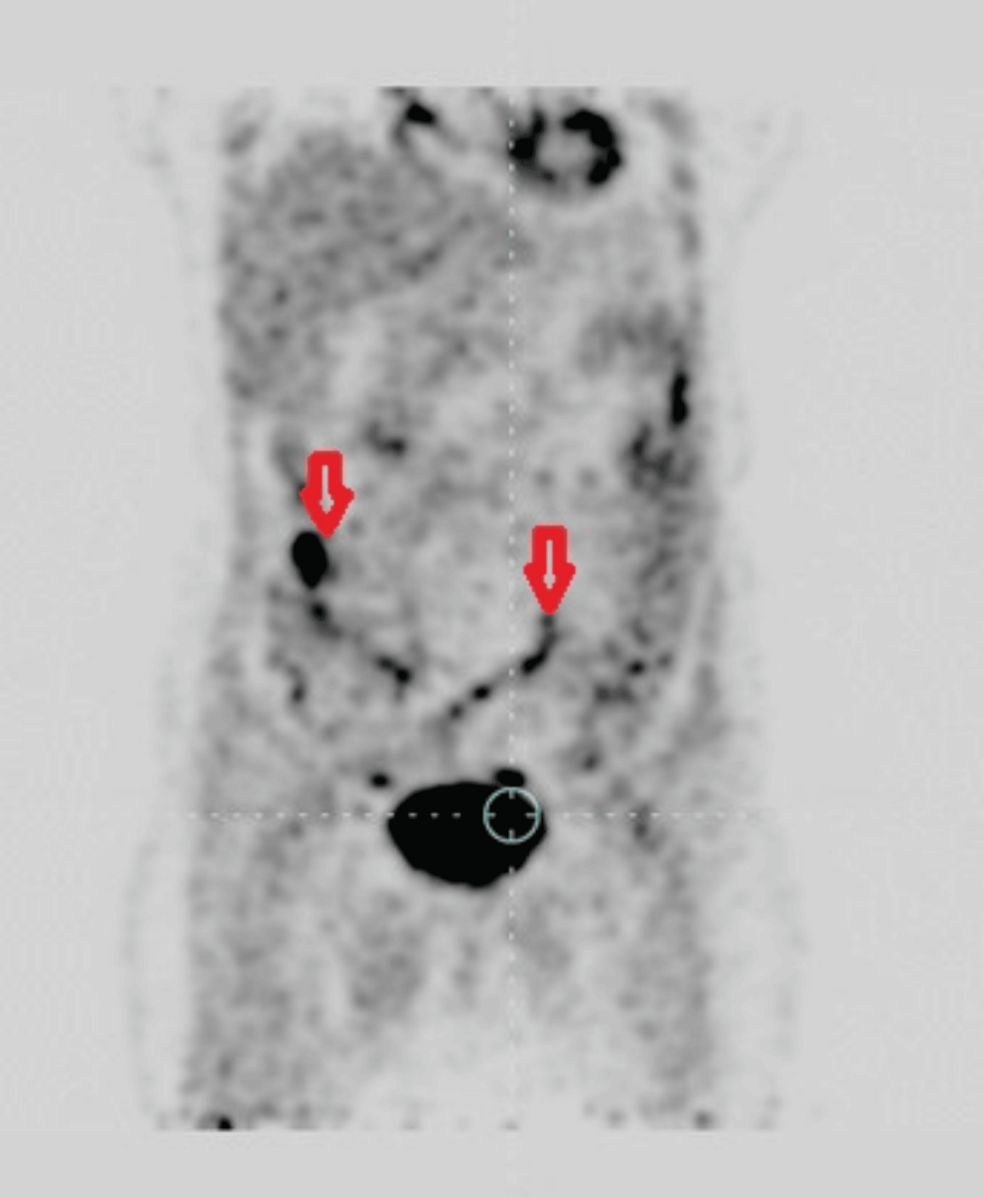
CECT photograph showing enlarged pelvic lymph nodes (Case 4). CECT: contrast-enhanced computed tomography.

Owing to its excellent contrast nature (T2-weighted image), the MRI showed sarcomatoid differentiation, a distinguishing feature from endometrial carcinoma. Fludeoxyglucose-18 positron emission tomography CT (FDG PET/CT) is a highly sensitive modality employed for the detection of regional and distant nodal metastasis. In our study, two patients (Cases 1 and 4) showed FDG PET/CT positive pelvic node metastasis (Figure 2).

**Figure 3:**
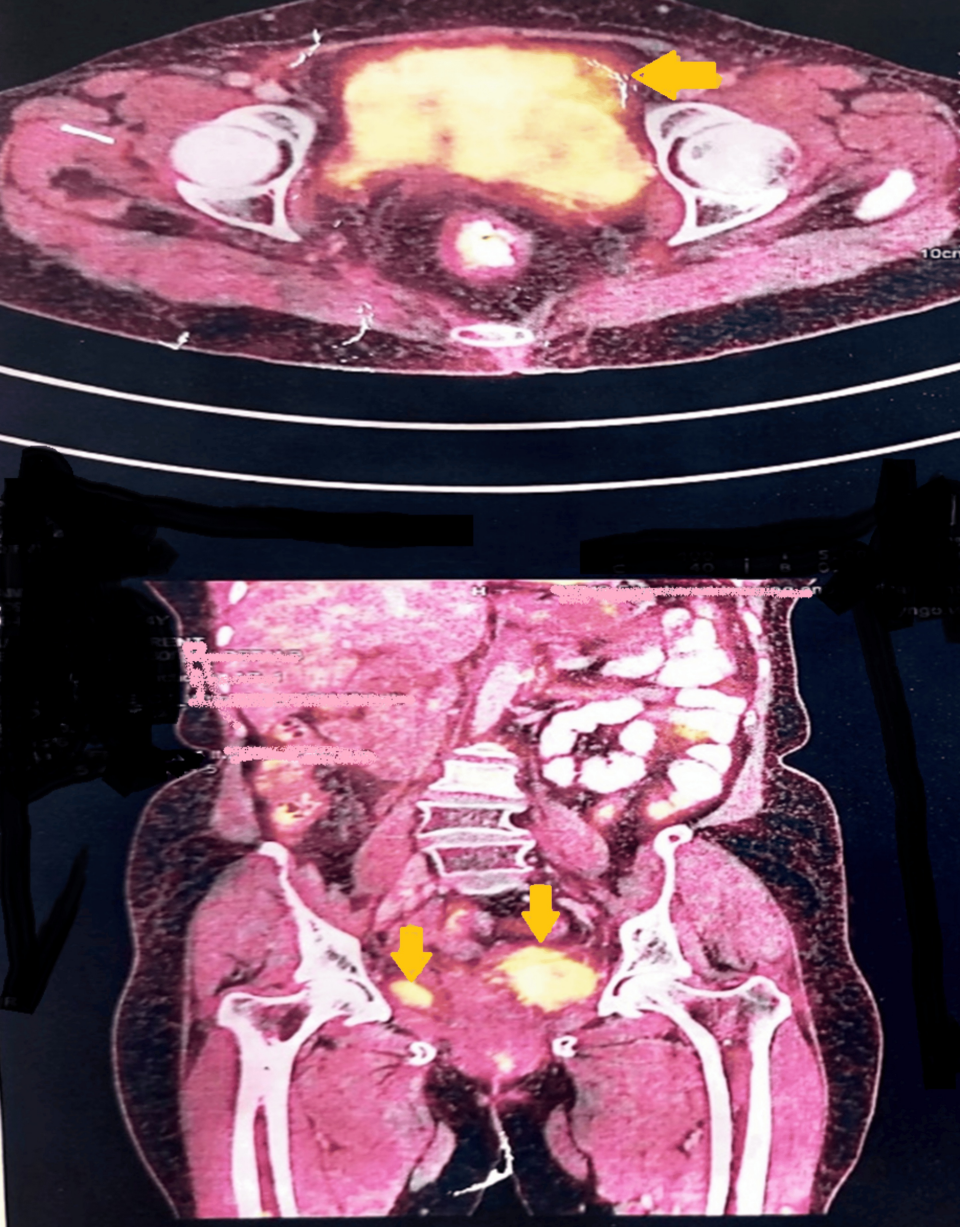
PET/CT image showing increased FDG uptake by the tumor (Case 4). PET/CT: positron emission tomography/computed tomography; FDG: fludeoxyglucose-18.

PET/CT showed a primary tumor and multiple metastatic areas showing increased FDG uptake in Case 4 (Figure 3).

Histopathological findings

**Figure 4:**
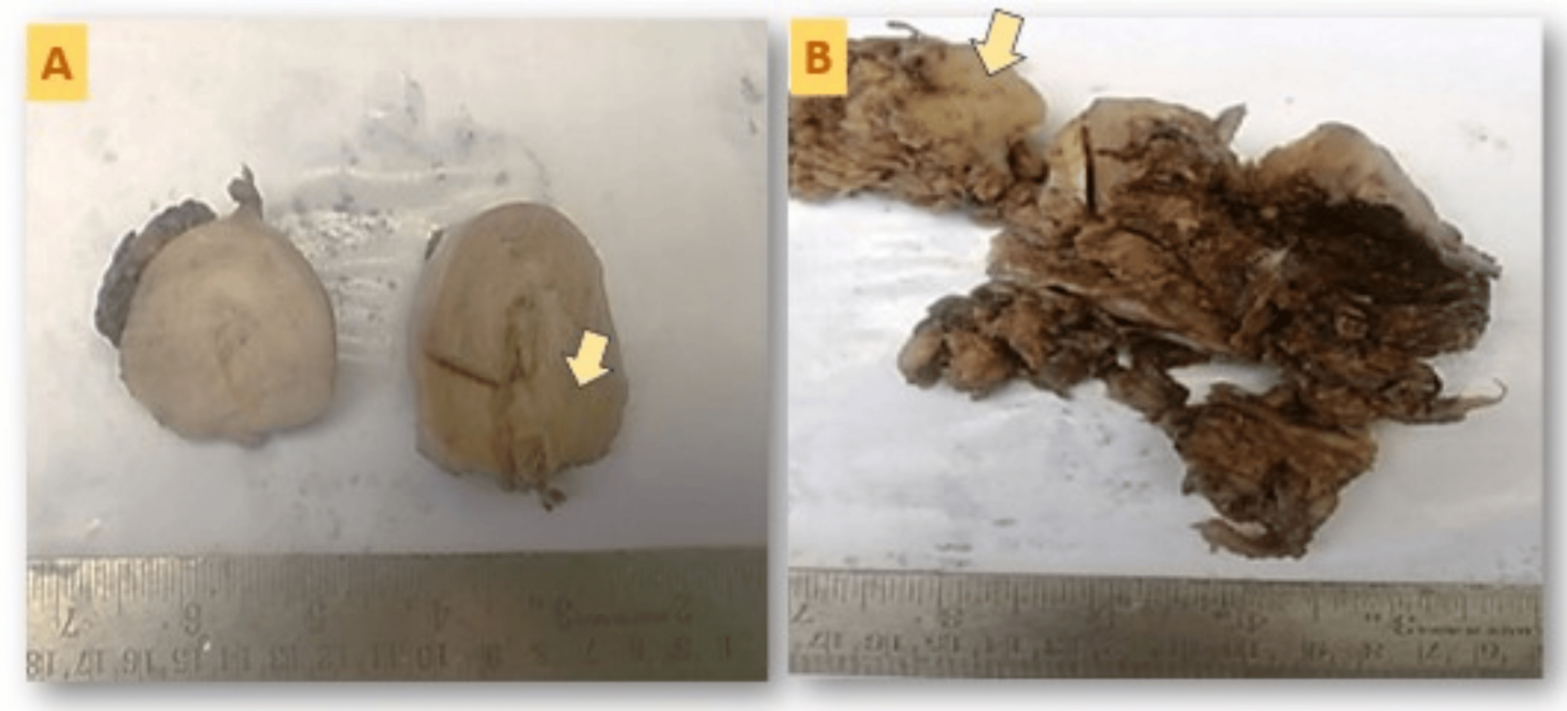
Gross images of cases. (A) Photomicrograph of the thickened uterine wall in Case 1; (B) photomicrograph of the ovary showing solid cystic areas in Case 4. The arrows showed a thickened tumor area.

Grossly tumor was limited to the myometrium and visible as a uniform thickening of the uterine muscle layer (Figure 4).

**Figure 5:**
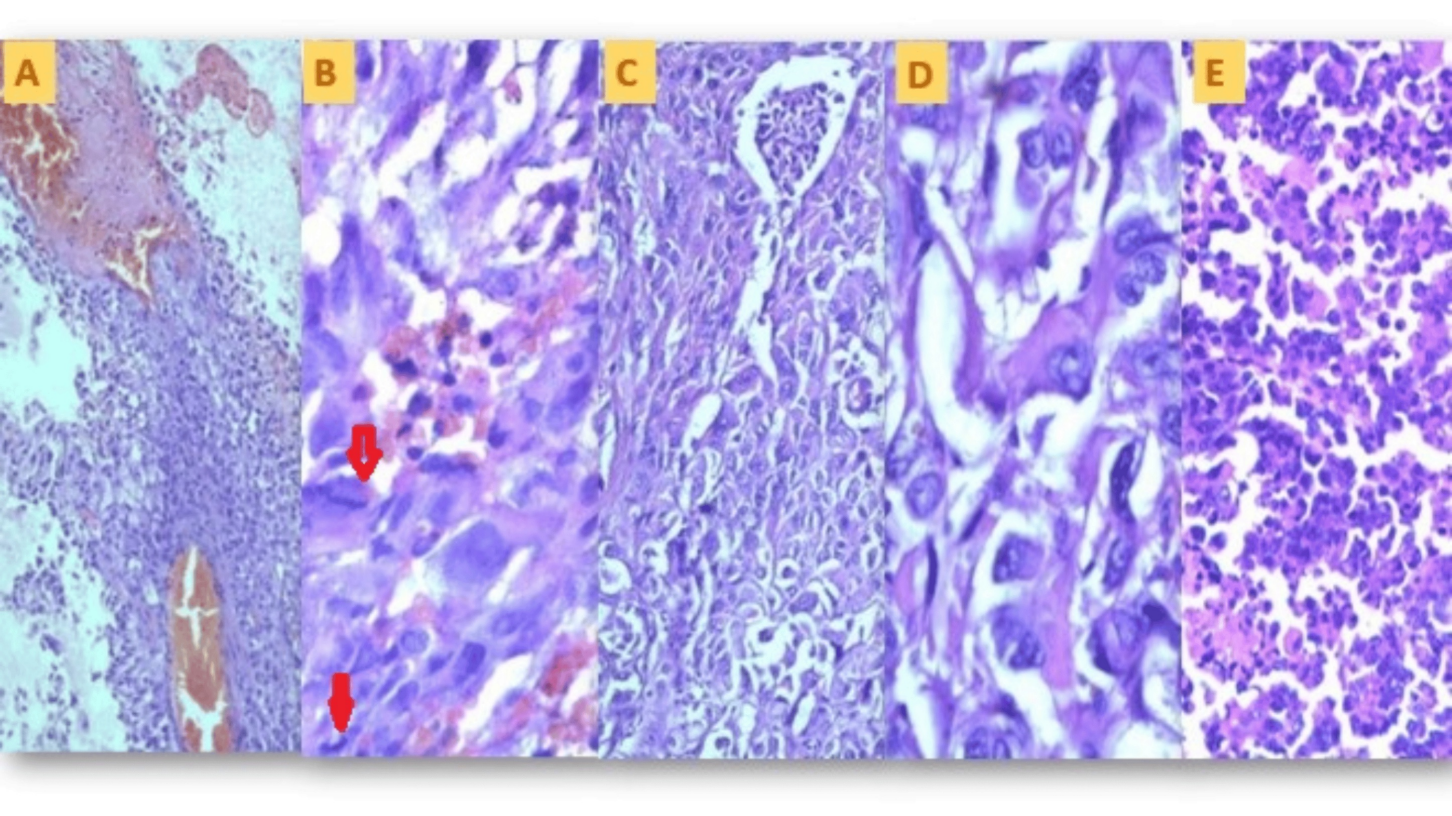
Photomicrographs of microscopic histology of cases. (A) Low-power (100×) photomicrograph of the tumor cells with hemorrhagic areas (Case 1); (B) high-power (400×) photomicrograph of the sarcomatous component (Case 1), with the arrows pointing to the atypical mitosis; (C) mid-power (200×) photomicrograph of the sarcomatous component (Case 1); (D) high-power (400×) photomicrograph of the sarcomatous component (Case1); (E) low-power (100×) photomicrograph of the tumor cells (Case 2); (F) low-power (100×) photomicrograph of the tumor cells (Case 2).

The tumor metastasized into the ipsilateral ovary, replacing the whole of the ovarian parenchyma in Case 1. The tumor exhibited a biphasic structure at the microscopic level, characterized by high-grade epithelial (carcinomatous) and spindle-shaped (sarcomatous) components. The carcinomatous component was found to be high-grade endometrioid type (Case 1) (Figures 5A-D). Case 2 also showed tumor cells of similar morphology (Figures 5E, 5F).

**Figure 6:**
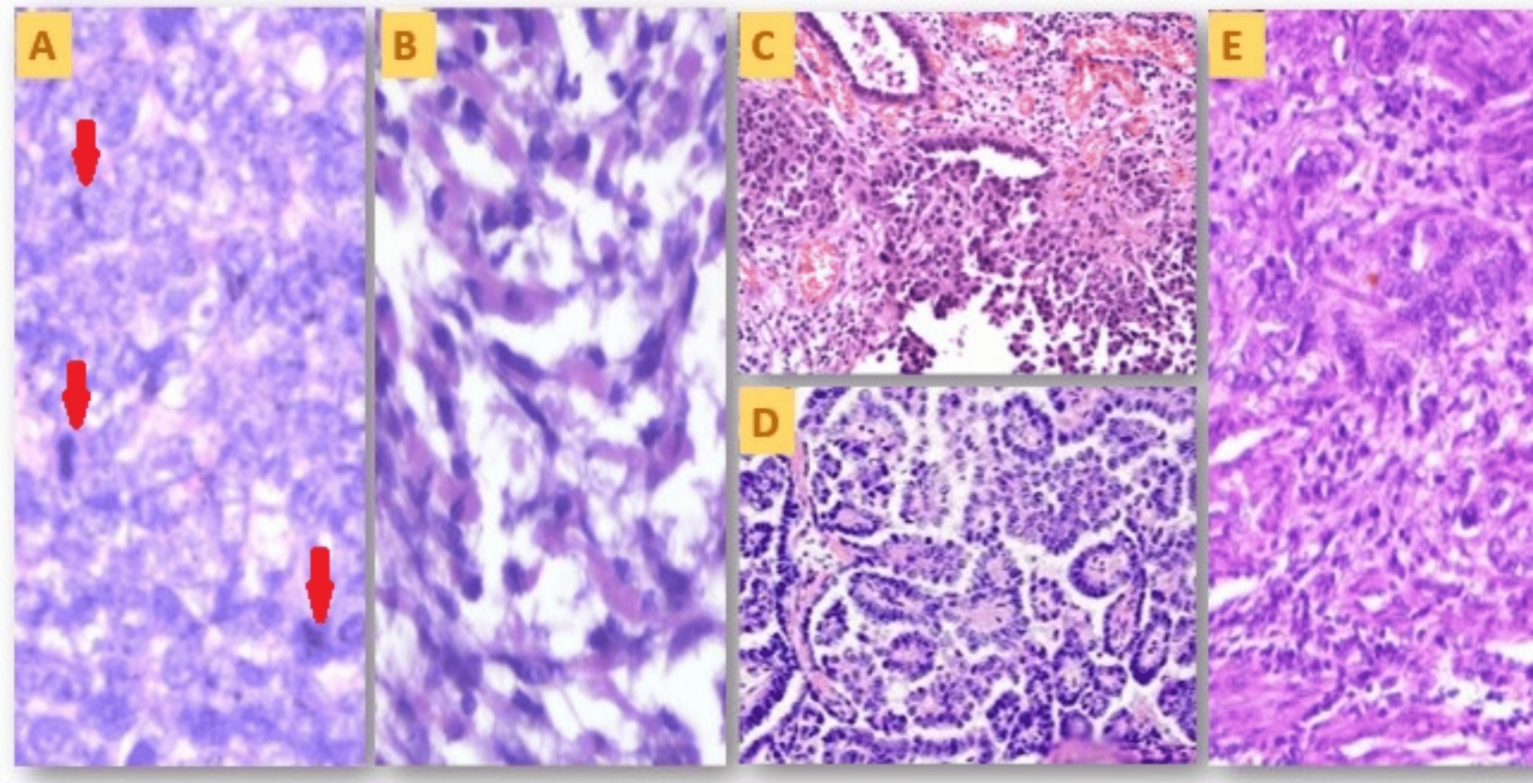
Photomicrographs of the microscopic histology of cases. (A) High-power (400×) photomicrograph of the epithelial component (Case 2), with the arrows showing frequent atypical mitosis; (B) high-power (400×) photomicrograph of the sarcomatous component (Case 2); (C) high-power (400×) photomicrographs of the epithelial component (Case 4); (D) high-power (400×) photomicrograph of the serous papillary epithelial component in the ovary (Case 3); (E) high-power (400×) photomicrograph of the epithelial component (Case 5).

The serous papillary kind is the most prevalent, as illustrated in Figure 6D. Occasional epithelial components also showed undifferentiated hybrid serous to endometrioid-type morphology (Figures 6C, 6E). The sarcomatous component was high-grade, showing highly pleomorphic dyscohesive spindle cells having a high nucleo-cytoplasmic ratio, coarse to clumped chromatin, and scant cytoplasm. Occasional heterologous elements, such as rhabdomyosarcomatous components, were intermixed (Case 2) (Figure 6B). Mitotic count was high, varying between 12/10 hpf and 22/10 hpf (Figure 6A). Necrosis was frequent but limited to focal areas, and no geographical large necrosis was seen.

IHC findings

**Figure 7:**
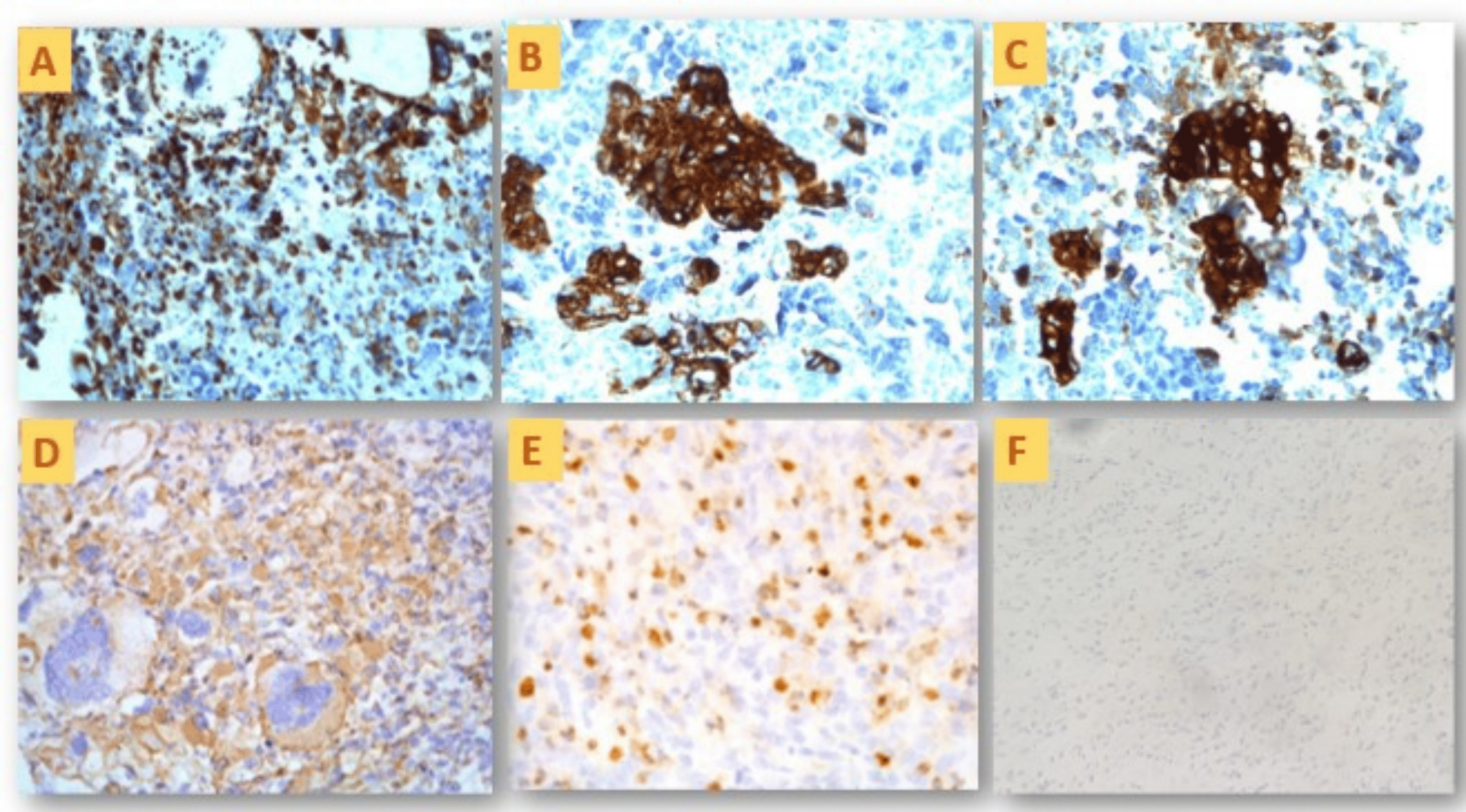
Photomicrographs of immunohistochemistry (IHC) expression of cases. (A) Vimentin positivity in the tumor cells (Case 1); (B) CK positivity in the epithelial tumor cells (Case 1); (C) EMA positivity in the epithelial tumor cells (Case 5); (D) CA-125 positivity in the tumor cells (Case 4); (E) myogenin positivity in the tumor cells (Case 2); (F) LCA negativity in the tumor cells (Case 6).

IHC was considered for the confirmation of morphological diagnosis and used for proper tumor typing. Extended IHC markers were used to exclude the differentials (Table 2). Pan-CK was positive in the tumor's epithelial component (Figure 7A), while vimentin was positive (Figure 7B) in high-grade sarcomatous components. EMA was positive in Cases 1, 5, and 6 (Figure 7C). Myogenin was positive in the heterologous rhabdomyosarcomatous component in Case 2 (Figure 7E).

WT-1 was positive for serous papillary cystadenocarcinoma in the right ovary. The serum CA-125 level was raised in Case 4, and there was immunopositivity in the ovarian tumor (Figure 7D). Negative IHC markers were equally important as they helped to rule out the morphological differentials. LCA was used to rule out any lymphoid malignancy (Figure 7F). Synaptophysin and chromogranin were utilized to rule out neuroendocrine malignancy, similarly HNM-45 for melanoma and CD-68 for undifferentiated sarcoma.

## Discussion

Our study described the clinical and histopathological characteristics, treatment outcomes, and overall survival characteristics of six cases diagnosed as malignant MMT or carcinosarcoma in a tertiary care center in northern India. The primary site of the lesion was the uterus in four cases, whereas the ovary was the site in the other two. Uterine carcinosarcoma is a malignant neoplasm that mimics clinically, most commonly high-grade endometrial carcinoma, as both present with the features of pain, bleeding, and a rapidly enlarging lump [12]. The overall five-year survival is approximately 33%-39% [13].

Previously, carcinosarcoma was categorized under the sarcoma group of tumors characterized by both epithelial and sarcomatous differentiations; however, further IHC and molecular studies demonstrated it to be carcinoma with sarcomatous metaplasia of monoclonal cancer cells [14]. The chief complaint in our study was also abnormal uterine bleeding followed by lower abdomen heaviness. Tirumani et al. [3] conducted a comprehensive review of uterine sarcoma and concluded that the prevalent differentials considered include high-grade endometrial carcinoma and its variants such as serous, endometrioid, undifferentiated carcinoma and malignant mesenchymal tumors such as leiomyosarcoma, adenosarcoma, and undifferentiated pleomorphic sarcoma. In this study, the same common differentials were also considered.

Kanthan and Senger [15] conducted a thorough review of the literature and concluded that most of the carcinosarcoma behaves like grade II endometrial carcinoma; however, higher chances of pulmonary metastasis, local spread, and peritoneal seedings were noted. This study also demonstrated ovarian metastases and peritoneal dissemination in Cases 1 and 2. Like another high-grade endometrial carcinoma, carcinosarcoma has a propensity to spread through transperitoneal, thus causing positive peritoneal washing along with omental, peritoneal, and adnexal metastasis. A series of carcinosarcoma cases reported that up to 19%-44% of women present with positive peritoneal washing at the time of diagnosis [16]. In our study, two instances were also diagnosed with positive peritoneal washing at the time of diagnosis.

Kajo et al. [17] reported a 53-year-old female with metastatic carcinosarcoma who died eight months post-diagnosis; similarly, in our investigation, two instances with peritoneal metastasis died after 15 days and 11 months post-diagnosis. Anupama et al. [18] performed a retrospective analysis including 20 carcinosarcoma patients over a five-year period and determined that 75% of the patients were classified as stage I and II. Patients with extrauterine presentation at the time of surgery had a survival duration of approximately nine months, but those with tumors confined to the uterus exhibited a superior overall survival of approximately 36 months. Our investigation demonstrated concordance, revealing that in two cases with peritoneal metastasis, the mean overall survival was around 10 months, whereas in cases with stage I and II tumors, survival was approximately two years and six months.

Additional studies indicated that prognosis predominantly relies on the extrauterine presentation at the time of diagnosis [19]. Surgery is the principal treatment approach, encompassing total abdominal hysterectomy, bilateral salpingo-oophorectomy, peritoneal or omental assessment, peritoneal cleaning, and excision of para-aortic and pelvic lymph nodes. Adjuvant chemotherapy, succeeded by external beam radiation, constitutes the conventional treatment protocol [20,21]. This study also included two stage IV instances that underwent chemotherapy and radiotherapy following surgical resections. Two cases of stage III and II underwent adjuvant chemotherapy following surgical resection, and two cases of stage I received surgical resection only.

Treatment and survival outcomes

Out of six cases included in this study, two patients with stage IV died after 15 days and 11 months after diagnosis. One patient of stage III received chemotherapy (taxane-platinum-based paclitaxel/carboplatin) and radiotherapy and died after six months of diagnosis. One patient of stage II received adjuvant chemotherapy and was alive three years post-diagnosis. In two stage I patients, only surgical resections were performed without chemotherapy or radiotherapy, and they are under close follow-up; both are alive after two years and one year post-diagnosis, respectively.

Strengths and limitations of the study

The uniqueness of the study is that it is a single-center study in North India, examining a rare tumor type in a specific population. Additionally, the current investigation documented clinical, radiological, histopathological, and IHC details, together with comprehensive follow-up data. The primary constraint of the study is the small sample size (n=6), which restricts the generalizability of the findings. The investigation was retrospective, resulting in poorly preserved imaging recordings, and recollection bias significantly influenced data collection. A multicentric investigation with a larger sample size would enhance the conclusions and validate the findings.

## Conclusions

Carcinosarcoma is a malignant and very aggressive tumor primarily originating in the uterus and less frequently in the ovaries. Previously, these tumors were classified as sarcoma, but recent studies proved them to be high-grade endometrial carcinoma with sarcomatous metaplasia. Clinical features are indistinguishable from high-grade endometrial carcinoma. An IHC panel is needed to differentiate morphological diagnosis and confirm the tumor type. In the current study, we have tried to highlight the clinical, radiological, and pathological features as well as recorded treatment protocols and survival outcomes of rare ovarian and uterine carcinosarcoma cases (n=6). This study provides insight into the importance of histological and IHC analysis in the early diagnosis of the disease to achieve better survival outcomes.
